# Perspective on the Structural Basis for Human Aldo-Keto Reductase 1B10 Inhibition

**DOI:** 10.3390/metabo11120865

**Published:** 2021-12-13

**Authors:** Francesc Xavier Ruiz, Xavier Parés, Jaume Farrés

**Affiliations:** 1 Center for Advanced Biotechnology and Medicine, Department of Chemistry and Chemical Biology, Rutgers University, Piscataway, NJ 08854, USA; 2 Department of Biochemistry and Molecular Biology, Faculty of Biosciences, Universitat Autònoma de Barcelona, Bellaterra, E-08193 Barcelona, Spain; xavier.pares@uab.cat

**Keywords:** aldo-keto reductase, aldose reductase, cancer, enzyme inhibitor, structure-based drug design

## Abstract

Human aldo-keto reductase 1B10 (AKR1B10) is overexpressed in many cancer types and is involved in chemoresistance. This makes AKR1B10 to be an interesting drug target and thus many enzyme inhibitors have been investigated. High-resolution crystallographic structures of AKR1B10 with various reversible inhibitors were deeply analyzed and compared to those of analogous complexes with aldose reductase (AR). In both enzymes, the active site included an anion-binding pocket and, in some cases, inhibitor binding caused the opening of a transient specificity pocket. Different structural conformers were revealed upon inhibitor binding, emphasizing the importance of the highly variable loops, which participate in the transient opening of additional binding subpockets. Two key differences between AKR1B10 and AR were observed regarding the role of external loops in inhibitor binding. The first corresponded to the alternative conformation of Trp112 (Trp111 in AR). The second difference dealt with loop A mobility, which defined a larger and more loosely packed subpocket in AKR1B10. From this analysis, the general features that a selective AKR1B10 inhibitor should comply with are the following: an anchoring moiety to the anion-binding pocket, keeping Trp112 in its native conformation (AKR1B10-like), and not opening the specificity pocket in AR.

## 1. Introduction

Aldo-keto reductases (AKRs) constitute a superfamily of NADP(H)-dependent, monomeric oxidoreductases, mostly cytosolic, catalyzing the reduction of carbonyl-containing compounds to their corresponding alcohols. In this case, 15 human AKRs have been described belonging to six different subfamilies: AKR1A, AKR1B, AKR1C, AKR1E, AKR6A, and AKR7A. There are three members of the human AKR1B subfamily, namely AKR1B1 (aldose reductase, AR), AKR1B10 (aldose reductase-like protein-1), and AKR1B15, which share 71% amino acid sequence identity and overlapping substrate specificities for aliphatic and aromatic aldehydes. AR is a ubiquitous enzyme and has been thoroughly investigated because it participates in glucose reduction under hyperglycaemia, being involved in the secondary complications of diabetes. This has elicited a long-lasting search for AR inhibitors (ARIs) as antidiabetic drugs. AKR1B10 has a very high catalytic efficiency for all-*trans*-retinaldehyde and a more specific tissue expression (mostly in the gastrointestinal, GI, tract), although it is overexpressed in several cancer types and skin diseases. AKR1B15 is likely a mitochondrial protein and its mRNA has been found in placenta, testis, skeletal muscle, and adipose tissue [1,2,3].

Luckily enough, an elevated number of high-quality crystallographic AKR structures (AKR1B1, 156; AKR1B10, 20) are available from the Protein Data Bank (PDB), some with a resolution higher than 1 Å, and many including ternary complexes with inhibitors. This makes AKR superfamily to be one of the best-known enzyme systems at atomic level with a well-established catalytic mechanism. AKRs share an (α/β)_8_ barrel core motif, also named TIM barrel after triose phosphate isomerase, a conserved metabolic enzyme. The (α/β)_8_ barrel is the most common fold among protein catalysts, appearing in approximately 10% of all known enzyme structures [4]. The active site of AKRs includes a conserved catalytic tetrad consisting of residues Asp43, Tyr48, Lys77, and His110 (AKR1B1 numbering). Tyr48, His110, Trp111 and the nicotinamide moiety of NADP^+^ define a geometrically rigid “anion-binding pocket” (ABP). The existence of this pocket was originally established from the structures of the complexes of AKR1B1 with citrate, cacodylate and glucose-6-phosphate. Inhibitors occupy this pocket with a negatively charged group, e.g., by using a carboxylate, hydantoin, or succinimide function. Three external and otherwise highly variable loops (named A, B and C) connecting residues 112–136, 216–227, and 298–310, respectively, contribute to protein plasticity, substrate specificity and inhibitor selectivity. Loop B acts as a safety belt upon cofactor binding and it is stabilized by a salt bridge between Lys21 and Asp216.

Adjacent to the AKR1B1 active site, a transient subpocket may exist which can be opened by induced fit, the so-called “specificity pocket” (SP), enabling a significantly enlarged active site. The SP is lined by Trp111, Thr113, and Phe122 from loop A, and Cys298, Ala299, Leu300, Leu301, Ser302, and Tyr309 from loop C. Its opening is generally driven by a second, usually hydrophobic, inhibitor moiety. To accomplish this opening, loop C, especially next to Leu300, shows high flexibility [5]. Trp111 occupies a privileged position as a hinge region between the ABP and the SP. In other AKRs, such as AKR1A1 (aldehyde reductase) and AKR1B15, the establishment of a similar pocket upon ligand binding appears rather unfavorable [5,6,7].

Here we carefully analyze the structural properties of AKR1B10-NADP^+^-inhibitor complexes comparing them with those of analogous AR structures. We emphasize the distinct structural conformers revealed upon inhibitor binding, which define additional binding subpockets. Finally, we provide some hints for structure-based drug design of more selective AKR1B10 inhibitors.

## 2. AKR1B10 Inhibition Strategies

Since the 1980s, AR has been deeply studied as a drug target [8,9] because it transforms cytosolic glucose into sorbitol (a reaction that AKR1B10 and AKR1B15 cannot perform [7,10]), though only under hyperglycemia. Despite many positive pre-clinical studies on ARIs, most clinical trial outcomes have been disappointing. The failure of ARIs as therapeutic agents has been mainly attributed to poor pharmacokinetic properties, lack of clinical efficacy, and/or unacceptable side effects. Most ARIs contain either a cyclic imide group, such as a spirohydantoin group or a spirosuccinimide group, or an acetic acid moiety. The carboxylic acid-containing inhibitors have lower in vivo efficacy, which has been attributed to the relatively low pKa value of the carboxyl group, thus causing ionization at physiological pH and an inability to cross cell membranes. Conversely, cyclic imides have higher pKa values and are only partially ionized at physiological pH, thus allowing to pass through cell membranes and therefore having better pharmacokinetic properties [6,11,12].

Recently, a novel approach using intra-site differential inhibitors against AR has been proposed. These inhibitors may act differentially on AR activity depending on the nature of the substrate, in such a way that they could interfere specifically with the transformation of some substrates while leaving the conversion of other substrates free to occur. This means that the damaging activity of AR (e.g., glucose reduction) could be diminished without compromising the detoxifying role of the enzyme. A few natural AR differential inhibitors from plant extracts have been reported [13,14,15].

Regarding the effect of ARIs against other enzymes (especially from the AKR superfamily), initially the only cross-inhibition target thoroughly analyzed had been human aldehyde reductase (or AKR1A1) [6]. Nevertheless, AKR1A1 presents notable differences in respect to AKR1Bs: (i) it lacks the hyper-reactive active site cysteine (Cys298 in AR) and the Nε of the imidazole ring of the active site histidine interacts with the amide side chain of the nicotinamide ring of NADPH; (ii) the size of loop C is nine residues longer than that of AKR1Bs, determining a rather distinct substrate specificity and inhibitor selectivity [1,5,6]. As explained above, AKR1B10 is in fact the closest enzyme to AR (sharing 71% amino acid identity), and we and others surmised that the lack of selectivity of ARIs could be a relevant factor contributing to their failure as pharmacological drugs [10,11,12]. Furthermore, AKR1B10 is now established as a promising cancer target (except for gastric cancers, where it is downregulated) [12,16], and the ubiquitously expressed AR can represent a problematic off-target, given its overall similarity with AKR1B10. Next, we will provide an overview of the different AKR1B10 inhibitor types in the context of the available three-dimensional structures of AKR1B10 deposited in the PDB (Table A1). To note that an exhaustive listing and description of AKR1B10 inhibitors is beyond the scope of this review. We refer the reader to the revisions of Huang et al. [17] and, more recently, Endo et al. [16,18] for further details.

### 2.1. AKR1B10 Reversible Inhibitors

**Figure 1:**
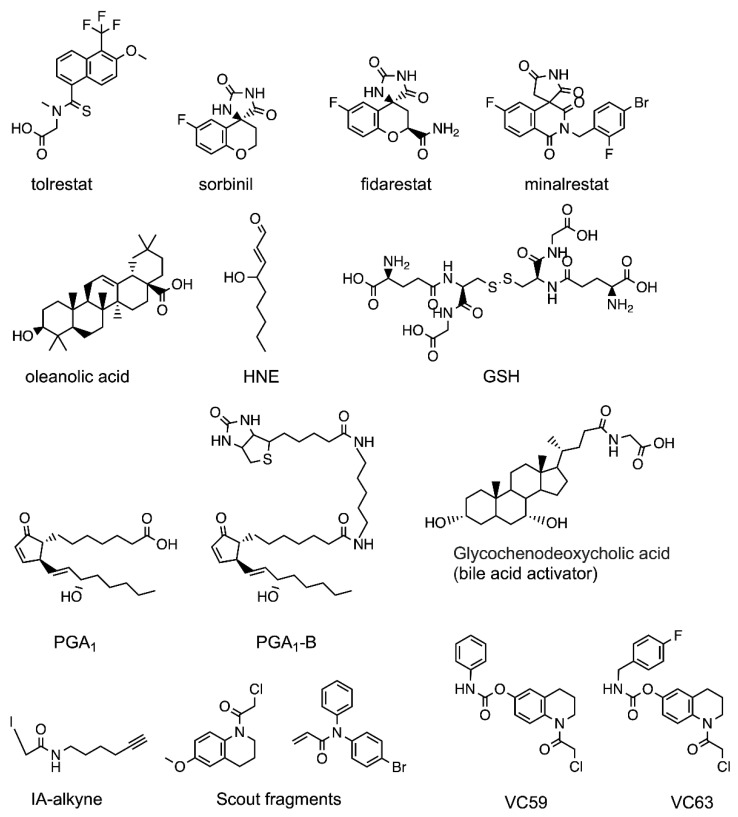
Molecular structures of some of the compounds cited in the manuscript. Molecular formulas were made with ChemDraw 19.0.0.26.

**Figure 2:**
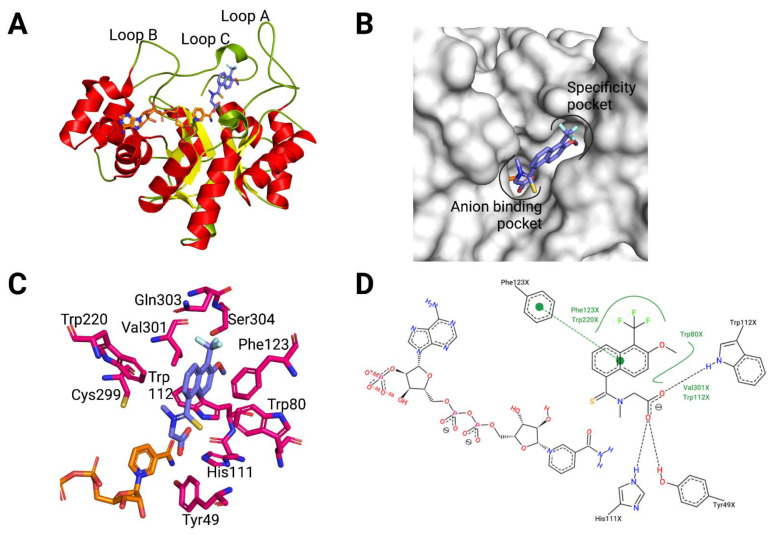
Overview of the AKR1B10 three-dimensional structure. (**A**) Crystal structure of AKR1B10 complexed with NADP^+^ (orange) and tolrestat (violet, PDB ID 1ZUA), with side view of the (α/β)_8_ barrel, with α helices in red, β sheets in yellow and loops in green. (**B**) Surface representation of the structure, indicating location of the anion-binding pocket (ABP) and specificity pocket (SP). (**C**) Atomic model describing interactions of the tolrestat molecule in the AKR1B10–NADP^+^–tolrestat complex. (**D**) 2D representation of the previous. Created with PyMoL 2.3.0. and in biorender.com.

The first AKR1B10 inhibitors described were in fact non-selective ARIs, e.g., tolrestat (Figure 1, [10,19,20]). In general, most of these ARIs belonged to the carboxylic acid type, while most cyclic imide ARIs tested (e.g., fidarestat, Figure 1) were poor AKR1B10 inhibitors [21,22], except for minalrestat ([23], Figure 1). Like ARIs, AKR1B10 inhibitors exploit the hydrophilic nature of the enzyme active site, containing the ABP. This pocket involves catalytic residues Tyr49 and His111, key residue Trp112 (AKR1B10 numbering) and the positively charged nicotinamide moiety of the cofactor NADP^+^ ([10,11], Figure 2). Therefore, all AKR1B10 inhibitors present a negatively charged or electronegative moiety that anchors them to the ABP and display an uncompetitive inhibition pattern despite binding to the same pocket than substrates [11,22,24]. This behavior is related to the conserved AKR kinetic mechanism, strictly ordered, with the cofactor binding first and leaving last: substrates are binding with higher affinity to the AKR-NADPH complex while inhibitors interact better with the AKR-NADP^+^ complex [1,25,26].

Hence, considering this anchoring moiety, we can broadly divide AKR1B10 inhibitors into two types: carboxylic acid- and non-carboxylic acid-containing inhibitors (hereinafter, CAIs and NCAIs, respectively). Section 3.2. will provide relevant examples and binding insights of each class whose structure in complex with the AKR1B10 holoenzyme (AKR1B10-NADP^+^) has been solved.

### 2.2. AKR1B10 Covalent Inhibitors

Despite its wide use in medicine (e.g., aspirin, penicillin, acetaminophen), there has been some reluctance in pursuing covalent inhibition in drug discovery until recently, because of off-target binding and potential toxicity. This tendency has reversed upon FDA-approval of several covalent drugs [27]. Accordingly, covalent inhibition of AR or other human AKR1s such as AKR1B10 is an unexploited strategy. Both AR and AKR1B10 possess a reactive cysteine (Cys298/Cys299, respectively) in their active site [22,28]. Nevertheless, there is a lack of thorough understanding of its in vivo role if any.

Indeed, Cys298 in AR is highly nucleophilic and can be reversibly or irreversibly modified by different reactive species such as nitric oxide (NO), 4-hydroxynonenal (HNE, Figure 1), or oxidized glutathione (GSH, Figure 1) in both recombinant protein and ex vivo models [29]. These modifications can reduce or increase the catalytic activity of AR, depending on the modifying moiety, and reduce its susceptibility to some non-covalent inhibitors, while increasing concentrations of the reduced cofactor NADPH protect Cys298 from modification [29,30]. This oxidated form of AR is called “activated AR” *versus* the “native AR” (with reduced Cys298). Balendiran and colleagues [29] generated the C298S AR mutant, a good surrogate of activated AR, and studied it biophysically. They solved the C298S AR holoenzyme structure (PDB ID 3Q67) and identified that Ser298 makes a hydrogen bond contact with Tyr209, restricting the flexibility of the mutant in comparison to the native holoenzyme, with Cys298 and lacking this interaction. We and collaborators have recently generated another useful surrogate of the activated AR by means of X-ray irradiation [31]. Its structure (PDB ID 6F8O) displays a similar interaction with Tyr209, and comparison to structures containing ARIs shows that the “locked” residue 298 in activated AR may cause steric hindrance, explaining the reduced inhibition of some ARIs against activated AR.

Likewise, Balendiran and colleagues [22,29] observed similar trends of activity affection and inhibitor reduced potency with C299S AKR1B10, while Shen and colleagues probed “native” AKR1B10 with reactive oxygen species (ROS), GSH and free cysteine accounting for similar effects as for AR. Considering that Tyr210 (Tyr209 in AR) is conserved, it seems that AKR1B10 in vivo may also be regulated by the redox state. Last, the crystal structure of the AKR1B10 holoenzyme with epalrestat (PDB ID 4JIH, [32]) presents a sulfenylated Cys299, probably due to the crystallization conditions, further supporting the redox regulation of this residue.

This long prelude is necessary to understand the potential and the limitations of such approach for AKR1B10 (and AR) covalent inhibition. The first covalent inhibitor of AKR1B10 was found by Pérez-Sala’s laboratory in 2011 [28]. Using a proteomics approach, they found in mice fibroblasts that AKR1B3 (a mouse ortholog of AR) and AKR1B8 (a close AKR1B10 mouse homolog), sharing some key features but diverging in others [33,34]), were covalently bound to PGA_1_-biotin (PGA_1_-B, Figure 1). Furthermore, they showed that AKR1B10 is forming adducts with PGA_1_-B through Cys299, and that PGA_1_ inhibited its activity on antitumoral drug doxorubicin in human lung adenocarcinoma A549 cells, preventing chemoresistance [28]. A follow-up study by the same research group [35] proved that AR is also reacting covalently via Cys298 with PGA_1_ (Figure 1) and showed that, for both AKR1B10 and AR, the adduct could be reversed by high concentrations of GSH. Inhibition assays with recombinant proteins showed IC_50_ values of 38 and 16 μM against PGA_1_, respectively [35,36].

More recently, the Cravatt laboratory has found an additional couple of covalent leads, VC59 and VC63, with IC_50_ ~1 μM in AKR1B10-transfected cell lysates (Figure 1). They have developed powerful chemical proteomics approaches to map Cys ligandability in mammalian cancer cell lines [37]. In the first work [37], they used a broadly reactive iodoacetamide alkyne (IA-alkyne, Figure 1) in lung cancer cell lines and identified three liganded proteins exclusive to KEAP1-mutant cells (KEAP1 is a negative regulator of the transcription factor NRF2, which in cancer cells induces expression of metabolic enzymes such as AKR1B10 to restore redox homeostasis). In the second study [38], they have developed a second type of broadly reactive (but less unspecific) electrophilic fragment (“scout” fragments, Figure 1) with the same purpose of mapping Cys ligandability. AKR1B10 is used as a proof-of-concept, and again Cys299 has been identified as a highly reactive Cys with the scout fragments. Next, they screened a panel of ~140 evolved analogues based on the scout fragments, obtaining the mentioned two leads.

A common feature of both types of covalent inhibitors is that their discovery involved screening with cell lysates, not living cells [28,38]. Surprisingly, the most potent lead, VC59, did not bind to AKR1B10 in living lung cancer cells [38]. The researchers found out that, in cell lysates, increasing concentrations of NADPH prevented reactivity of Cys299. They argued that in living cells AKR1B10 is fully saturated with NADPH, which is expected according to the literature [12,39]. Balendiran and colleagues [29] observed that C298S AR binding to NADPH was diminished in comparison to wild-type AR, while NADP^+^ was unaffected. Thus, the polarity of the mutated and “locked” Ser298 side chain is likely to be less compatible with the NADPH complex compared with that with NADP^+^. Thus, the unfavourability of such a complex may prevent the reactivity of Cys298 (or Cys299 in AKR1B10). Hence, this warrants further research and consideration of both “native” and “activated” forms for drug discovery campaigns against both enzymes, and screening of compounds in living cells in different possible physiological and pathological redox scenarios.

### 2.3. Potential for AKR1B10 Catalytic Activators

AKR1B10 has a key role in protecting the GI tract from lipid peroxides and reactive aldehydes, and its expression is decreased in GI cancers [2,16]. Thus, finding small molecule activators of its activity could potentially be beneficial in both precancerous and cancerous lesions of the GI tract in which AKR1B10 downregulation has been observed. Indeed, some inroads into small molecule enzyme activators have been made through activity-based protein profiling or high throughput screening, including aldehyde dehydrogenase 2 [40], glucose-6-phosphate dehydrogenase [41] and serine hydrolase LYPLAL1 [42].

In this regard, Endo and colleagues reported that various bile acids (Figure 1) activated rat AKR1B14 catalytic activity [43]. Through a combination of kinetics, mutagenesis, and structural analyses, they identified that the likely mechanism of activation is acceleration of NADP^+^ dissociation, i.e., the rate-limiting step of the reaction catalyzed by AKR1Bs. This was surprising because most AKR1B and AKR1C enzymes are inhibited by bile acids [44,45]. However, His269 in AKR1B14 (a lysine in AKR1B10 and in most AKR1Bs apart from AKR1B15, [7]) was identified as a key residue for activation. Since the molecular basis for activation in AKR1B14 is well defined, and the differences with AKR1B10 are minimal, it is possible to envisage that a focused library of bile acid derivatives could help find the specific AKR1B10 activators.

## 3. What We Have Learnt from 3D Structures

The long-standing interest in AR is also reflected in the impressive number of three-dimensional structures of the holoenzyme by itself and in complex with many inhibitors (https://www.rcsb.org/uniprot/P15121, accessed on 8 December 2021), starting from the crystal structure of pig aldose reductase solved in 1994 [8]. Of note it is also the availability of over 30 structures with a resolution of 1 Å or higher, including the record resolution (0.66 Å) for a structure of a macromolecular entity over 25 kDa, the complex of AR holoenzyme with carboxylic ARI IDD594 (PDB ID 1US0 and [46]). Such detail level allowed identification of the protonation states of the residues involved in inhibition and catalysis, and it was later complemented by a joint X-ray/neutron crystallography structure that elucidated the catalysis and inhibition mechanisms of AR in extraordinary detail [47].

As explained for AKR1B10 inhibitors, the determination of structures of the AKR1B10 holoenzyme by itself and in complex with many inhibitors (20 structures, please see https://www.rcsb.org/uniprot/O60218, accessed on 8 December 2021, Table A1) had to wait a bit more than a decade (PDB ID 1ZUA and [10]) and provides a fair number of complexes containing CAIs and NCAIs, which will be addressed below.

### 3.1. AKR1B10 Structure: Overview and Specific Features

The first three-dimensional structure solved for AKR1B10, the ternary complex of AKR1B10/NADP^+^/tolrestat (PDB ID 1ZUA, Figure 2), was elucidated in a joint work by the groups of Parés/Farrés and Fita, and is yet the one with the highest resolution (1.25 Å). It illustrates the paradigm of a non-specific ARI binding to AKR1B10, with only positions 301 and 303 differing between AR and AKR1B10 for residues interacting with the compound [10]. The structure showed the (α/β)_8_ TIM barrel topology characteristic of the AKR superfamily, with the NADP^+^ cofactor bound in the interior of the barrel in an extended conformation (Figure 2A,B). Protruding from the barrel core, loops A (residues 112–136), B (residues 216–227) and C (residues 299–310), the most divergent in AKRs and conferring substrate specificity (Figure 2B), are forming the “lid” of the active site. Tolrestat interacts—through its carboxylic acid (CA) moiety—with the anion-binding site residues Tyr49, His111 (both along with Asp44 and Lys78 form the catalytic tetrad), and Trp112, near the positively charged nicotinamide ring of the cofactor (Figure 2C,D). The methoxy-trifluoromethyl-naphthalen moiety of tolrestat is lined by residues at the base of loops A and B (Trp112, Phe116, Phe123, Trp220) and by loop C (Cys299, Val301, Gln303). By comparison to AR, the latter pocket has also been named as “specificity pocket” (SP, Figure 2C).

**Figure 3:**
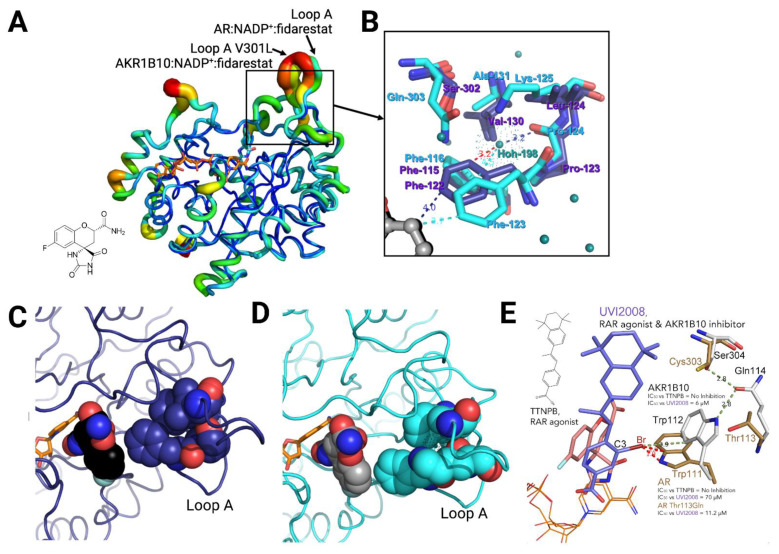
Specific features of AKR1B10 three-dimensional structure. (**A**) AR:NADP^+^:fidarestat and V301L AKR1B10:NADP^+^:fidarestat complexes in cartoon representation in B-factor putty mode to reflect the thermal flexibility of the structure (red: high B-factor to blue: low B-factor). (**B**) The zoom of part of the loop A region is an atomic model with color coding as follows: AR:NADP^+^:tolrestat (PDB ID 2FZD) in orange, AR:NADP^+^:fidarestat (PDB ID 1PWM) in deep blue, V301L AKR1B10:NADP^+^:fidarestat (PDB ID 4GAB) in cyan, with distances in the same color as the protein (or in red if they represent a short contact), water molecules in magenta for AR and in deep teal for the V301L AKR1B10 complex, and fidarestat is in black and gray, respectively. HOH-198 is also represented with dots to provide an idea of its size; (**C**) AR:NADP^+^:fidarestat complex and (**D**) V301L AKR1B10:NADP^+^:fidarestat complex represented in cartoon tube, the cofactor in orange sticks and fidarestat (black and gray, respectively) and key residues shown in space-filling model. (**E**) Superimposition of the AKR1B10 holoenzyme-UVI2008 complex (PDB ID 5M2F) with AR holoenzyme-fidarestat complex (PDB ID 1PWM). AKR1B10: white, AR: brown, NADP^+^: orange, UVI2008: violet, fidarestat: light pink. C3 halogen addition to *pan*-RAR agonist TTNPB enables AKR1B10 selectivity, facilitated by AKR1B10 Trp112 native conformation. Inhibition data provided for both complexes and AR Thr14813Gln, mutant disrupting the hydrogen bond network composed of residues 112, 114 and 304 stabilizing the native conformation. Panels (**A**–**D**) were adapted from Ref. [18]. Panel E was adapted from Ref. [48]. Created with PyMoL 2.3.0. and in biorender.com.

The determination of the structures of the AKR1B10 holoenzyme, alone and in complex with other ARIs and with specific AKR1B10 inhibitors, has allowed to identify two key differences between AR and AKR1B10 (see Table A1 for detailed information). The first corresponds to the different conformation of Trp112, in comparison to Trp111 in AR, in the holoenzyme by itself and with specific AKR1B10 inhibitors such as UVI2008 (Figure 3E). This conformation, which has been named as “native” or “1B10-like conformation”, is perpendicular to the “flipped” or “AR-like conformation”, observed in the case of AKR1B10/NADP^+^/tolrestat and complexes with other ARIs. As reported by us and the Hu’s laboratory [32,48,49], AKR1B10 adopts the native Trp112 conformation through a hydrogen bond network involving Gln114 and loop C residue Ser304 (Figure 2D). In AR, with Thr113 and Cys303, respectively, this network cannot be established. In addition, AR Trp111 conformation is always locked through a hydrophobic interaction with Leu300 (or an ARI opening the SP) [50].

The second main difference between AKR1B10 and AR is again involving one of the external loops. When comparing any of the AKR1B10 and AR structures, it is consistently observed, as derived from the thermal B factors, that loop A in AKR1B10 is much more mobile than in AR (Figure 3A). Furthermore, AKR1B10 presents a larger and more loosely packed loop A subpocket (LAS) than AR, with consistently observed crystallographic water molecule(s) trapped within (in 10 out of the 20 structures, Table A1, and Figure 3B–D). This subpocket in AR is normally absent and flanked on the sides by loop C Ser302 and loop A Phe122. In the case of AKR1B10, the flanking residues cannot come as close as in AR, due to the presence of the bulkier Gln303 side chain, resulting in an additional opening of ~2 Å of Phe123 side chain. In addition, in AR, Phe115 (Phe116 in AKR1B10), Leu124 (Lys125 in AKR1B10) and Val130 (Ala131 in AKR1B10) stack and make the pocket more hydrophobic, locked, and compact, being unable to allocate any water molecule without clashes (Figure 3B). On the contrary, in AKR1B10, the occupation of this subpocket and the capability of displacing the buried water(s) (see Table A1) are important for inhibitor binding and selectivity (discussed below) [21,50,51].

### 3.2. Structural Bases for AKR1B10 Selectivity

Aside from the difference in the active site region due to Trp112 unique conformation and the specific and imperfectly hydrated LAS, our biophysical and computational studies [21,50,51,52] have shown that AKR1B10 has different conformational landscape, hydration, and electrostatic properties than AR. In the previous sections, we have introduced the inhibitor types and the specific structural features of AKRB10. In this section, we will elaborate on what are the requirements for potent and selective AKR1B10 inhibitors, through a careful look to the different conformations of the holoenzyme upon their binding.

**Figure 4:**
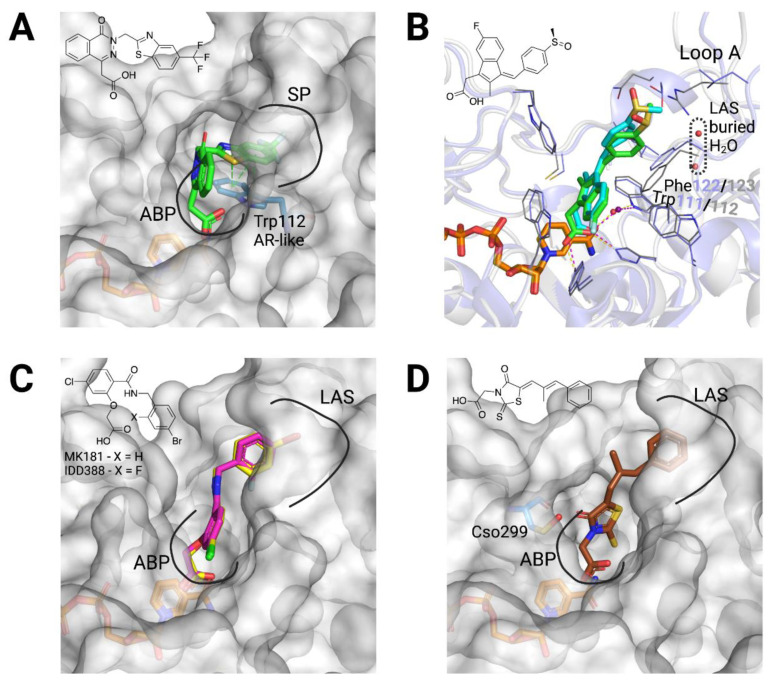
Structural bases for AKR1B10 selectivity (1). (**A**) Representation of the AKR1B10 (surface)-NADP^+^ (orange)-zopolrestat (green) structure (PDB ID 4JII), with anion binding pocket (ABP), specificity pocket (SP) and Trp112 conformation indicated. (**B**) Superposition of the AR-NADP^+^-sulindac complex (PDB ID 3U2C, violet, yellow sticks and cyan, respectively) and of AKR1B10-NADP^+^-sulindac complex (PDB ID 4WEV, white, orange and green, respectively), with loop A subpocket (LAS) buried water molecules, residues Trp111/112 and Phe122/123 and loop A indicated. Adapted from Ref. [46]. (**C**) Representation of the AKR1B10 (surface)-NADP^+^ (orange)-MK181/IDD388 (yellow/pink) structures (PDB ID 5LIK and 5LIU), with ABP and SP indicated. (**D**) Representation of the AKR1B10 (surface)-NADP^+^ (orange)-epalrestat (brown) structure (PDB ID 4JIH), with ABP, SP and Cso299 (S-hydroxycysteine 299) indicated. Since epalrestat was just partially solved, it has been docked manually. 2D molecular structures of inhibitors are also depicted. Created with PyMoL 2.3.0. and in biorender.com.

Regarding CAIs solved in complex to AKR1B10 holoenzyme (Table A1), several of them are also ARIs and bind AKR1B10 very similarly to AR, although with some exceptions. Tolrestat binding has already been considered in the previous section. Zopolrestat is also opening the SP in AKR1B10 analogously to in AR, through a π-π stacking interaction with Trp112 (Figure 4A). Sulindac, a non-steroidal anti-inflammatory drug (NSAID) previously reported to inhibit cyclooxygenase-2 (COX-2), AR and AKR1C3 [52,53], displays a mode of binding essentially equivalent in the two enzymes, stacking towards the base of loop A. However, the stacking interaction is different given by Phe122/Phe123, and two buried and ordered water molecules are present in the LAS in AKR1B10 but not in AR (Figure 4B). Regarding the mentioned exceptions, IDD388 and MK181 are known to open the SP in AR similarly to zopolrestat [54], but in AKR1B10, instead, they occupy the LAS bound in an extended conformation (Figure 4C). Lastly, epalrestat, in both structures with AR and AKR1B10 (PDB ID 4JIR and 4JIH, respectively), has not precise coordinates for the phenyl moiety and part of the linker to the CA moiety. Despite this, in AR, with no open LAS in the structure, it is expected that epalrestat may bind in a similar way as sulindac. Meanwhile, in AKR1B10, we can manually model epalrestat with its phenyl moiety occupying the LAS (Figure 4D).

**Figure 5:**
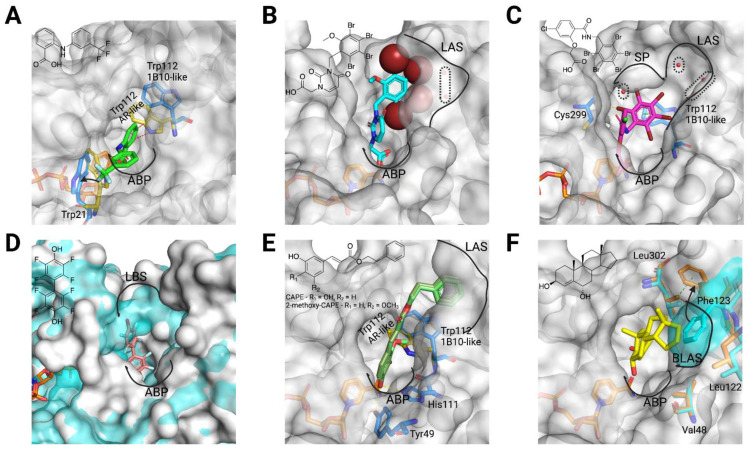
Structural bases for AKR1B10 selectivity (2). (**A**) Representation of the AKR1B10 (surface and blue)-NADP^+^ (orange)-flufenamic acid (green) structure (PDB ID 4I5X), with ABP, Trp21 and Trp112 conformations indicated. The last two are compared with the equivalent in the AKR1B10 holoenzyme structure (gold, PDB ID 4GQG). Short contacts between the inhibitor and Trp112 in AR-like conformation (in the holoenzyme structure) indicated with red dashed lines. (**B**) Representation of the AKR1B10 (surface)-NADP^+^ (orange)-JF0049 (cyan, Br atoms as spheres) structure (PDB ID 4XZL), with ABP and loop A subpocket (LAS) and its buried water molecules indicated. (**C**) Representation of the AKR1B10 (surface and blue)-NADP^+^ (orange)-MK204 (pink) structure (PDB ID 5LIY), with ABP, SP, Trp112 conformation, Cys299 and buried water molecules in SP and LAS indicated. (**D**) Representation of the AKR1B10 (surface and blue)-NADP^+^ (orange)-JF0064 (light pink) structure (PDB ID 4ICC), with ABP and loop B subpocket (LBS) indicated. The protein surface is compared to the equivalent in the AKR1B10 holoenzyme structure (blue surface, PDB ID 4GQG), showcasing the LBS opening induced by inhibitor binding. (**E**) Representation of the AKR1B10 (surface and blue)-NADP^+^ (orange)-CAPE/2-methoxy-CAPE (khaki/green) structure (PDB ID 4GQ0/manually docked), with ABP, LAS, Trp112 conformation, Tyr49 and His111 indicated. Trp112 conformation is compared with the equivalent in the AKR1B10 holoenzyme structure (gold, PDB ID 4GQG). Short contacts between the 2-methoxy-CAPE and Trp112 in AR-like conformation (in the holoenzyme structure) indicated with red dashed lines. (**F**) Representation of the AKR1B10 (surface and orange)-NADP^+^ (orange)-Androst-4-ene-3β,6α-diol (**3a**, yellow) structure (PDB ID 5Y7N), with ABP and base of loop A subpocket (BLAS, with its lining residues) indicated. The protein surface is compared to the equivalent in the AKR1B10 holoenzyme structure (blue surface and sticks, PDB ID 4GQG), showcasing the BLAS opening induced by inhibitor binding and involving a conformational change of Phe123. The last is stabilized in the current structure by a stacking contact with Leu302, indicated by a green dashed line. 2D molecular structures of inhibitors are also depicted. Created with PyMoL 2.3.0. and in biorender.com.

There are several CAIs, that are selective AKR1B10 inhibitors, for which the enzyme-NADP^+^-inhibitor structure has been solved (Table A1). Flufenamic acid is a NSAID and specific AKR1B10 inhibitor vs. AR but also inhibiting COX-2 and AKR1C3 [53]. Interestingly, in AKR1B10, it binds the holoenzyme with the aryl moiety stacking against Trp21 (Trp20 in AR), in a small loop near the active site. The selectivity is due to the steric clash that Trp111 in AR (always in flipped position) would have with the benzoic acid moiety of the inhibitor, and that the native Trp112 position avoids in AKR1B10 (Figure 5A).

The other two selective CAIs solved in complex with AKR1B10 holoenzyme, JF0049 and MK204 (Figure 5B,C), have in common polybrominated aryl moieties that are too bulky to fit within the SP of AR [50,51]. Nevertheless, they interact differently with AKR1B10. The aryl moiety of JF0049 is having a tight fit with the LAS, and we also observed an enthalpic signature by isothermal titration calorimetry upon its binding, consistent with the displacement of the water molecule trapped in the LAS in the holoenzyme structure. Indeed, the LAS presents just one or two ordered water molecules, but it is likely that other disordered and mobile water molecules are present. While their release should not contribute significantly to a large entropy gain, the new hydrogen bonds they will form with other water molecules in the bulk phase may add a significant enthalpic benefit [50].

On the other hand, MK204, with one additional bromine (Br) substituent in the aryl moiety and a three-atom linker between the CA and aryl moieties, was studied in the context of a series with increasing number of Br atoms in the aryl moiety of compounds with identical CA moiety and linker [51]. We observed that the three bulkier ligands can fit nicely into a novel AKR1B10 binding site conformer, mainly through a stacking interaction with the Trp112 native (but not the flipped) conformation (Figure 5B). Computational studies paired with the structures allowed us to surmise that ligand binding in this novel pocket requires a very hydrophobic aryl moiety able to displace unfavorable water molecules (accounting for a high desolvation penalty) observed in structures with less Br substituents than MK204 [51]. Furthermore, the latter (but not the other congeners) establishes a strong halogen bond with the main chain carbonyl of Cys299.

Regarding NCAIs solved in complex to AKR1B10 holoenzyme (Table A1), fidarestat and sorbinil (Figure 1), both cyclic imide ARIs, display an almost identical binding to the two enzymes (Figure 3A), not opening the SP but with a flipped Trp112. Next, we screened a library of synthetic polyhalogenated compounds lacking the usual CA or cyclic imide moieties (in collaboration with Biomar Microbial Technologies) and discovered JF0064, a *pan*-inhibitor against human AKR1B (in order of potency, inhibiting AKR1B15 > AR > AKR1B10 [7,11]) with a new anchoring moiety. We determined K_i_ values and complexes with AR and AKR1B10 holoenzymes, identifying it as a non-competitive inhibitor where the acidic hydroxyl group is binding the ABP, again not opening the SP but with a flipped Trp112. Of note is that JF0064 binding triggers a slight opening of loop B (loop B subpocket, or LBS), the only instance in which this has been observed in AKR1B10 structures (Figure 5D and Table A1). Chatzopoulou and colleagues [55], in an unrelated manner, developed a 2-fluoro-4-(1*H*-pyrrol-1-yl) phenol scaffold inhibiting AR, that showed improved membrane permeation, in line with our in vitro data predicting better pharmacokinetic properties for JF0064 and potential congeners [11].

A great number of selective CAIs and NCAIs was developed in the period from 2010 to 2015 [17,18]. Most of them are: (i) long aliphatic unsaturated compounds with terminal aryl moieties (caffeic acid derivatives, retinoids, etc.), or (ii) steroids. All these large inhibitors fit better the larger and more malleable (plastic) AKR1B10 active site and “lid” region (constituted by the three external loops A, B, and C), opposite to the snugger AR counterpart. We will address three of these compounds solved in complex with the AKR1B10 holoenzyme that illustrate the mechanistic bases of selectivity.

Regarding the first group, the Hu’s laboratory determined the structure for the NCAI lead caffeic acid phenethyl ester (CAPE) [49]. As we observed with JF0064, an acidic hydroxyl of the ligand is hydrogen-bonded to Tyr49 and His111 (Figure 5E) and Trp112 adopts the native conformation. CAPE would be compatible with the flipped conformation, but not CAPE derivatives with a 2-methoxy group in the catechol moiety (Figure 5E), which would clash with that conformation of Trp112 and have extraordinary selectivity for AKR1B10. This is similar to what we observed with UVI2008 (Figure 2D). Both compounds have aryl moieties that occupy the LAS (Figure 2D and Figure 5E).

Regarding triterpenoid inhibitors, such as oleanolic acid (Figure 1), molecular docking suggested that they would interact in a similar fashion as CAPE or UVI2008. The last AKR1B10 structure determined so far (PDB ID 5Y7N) is the first and only that contains a steroid inhibitor (an NCAI derivative from 5β-cholanic acid, androst-4-ene-3β,6α-diol (3a), [56]). While inhibition studies have been reported, the structure has not been published in a peer-reviewed journal. Complex with compound 3a shows a surprising feature: Phe123 is displaced inwards blocking the entry of the LAS and stacking against the side chain of Leu302, opening a novel subpocket that we name base of loop A subpocket (BLAS), delimited by Phe123, Leu122 and Val48 (Figure 5F). It should be of interest determining a structure of the AKR1B10 holoenzyme with oleanolic acid to see whether the latter binds similarly to 3a, in the BLAS, or it can open the LAS.

## 4. Conclusions

Different protein conformers may contribute to inhibitor selectivity against AKR1B10 *versus* AR. Due to the flexibility of the AKR1B10 active site and the existence of transient opening subpockets, the exact inhibitor-AKR1B10 interactions might need to be determined on a case-by-case basis using crystallographic methods. Upon close examination of the crystallographic structures of AKR1B10 with various inhibitors, distinct structural conformers were revealed. Here we summarize the general features that a selective AKR1B10 inhibitor should comply with:

(i) An anchoring moiety: Common to ARIs, an AKR1B10 inhibitor must have an anchoring moiety, with a carboxylic acid or an acidic hydroxyl as best choices. Cyclic imides, without the addition of an aryl moiety for binding to either the SP or the LAS (as it may be with minalrestat), are poor AKR1B10 inhibitors.
(ii) Keeping Trp112 in its native conformation (AKR1B10-like): Substituents or ligand conformations that are not compatible with the Trp112 flipped (AR-like) conformation—e.g., flufenamic acid, UVI2008−, and/or aryl moieties that provide an “optimal filling” of the LAS are required for specificity. That is to displace the buried water molecule(s) in the LAS, an adequate shape complementarity, and to have interactions that are more favorable that those in the bulk water [50,51].
(iii) Not opening the SP in AR: Another recurrent feature of selectivity for AKR1B10 over AR, is the inability of an inhibitor to induce the opening of the SP of AR, which normally occurs in ligands with a bulky aryl moiety as in JF0049 or MK204. This can be observed in Table A1, as, in the solved structures of AKR1B10 holoenzyme with inhibitors, no specific AKR1B10 inhibitor is able to open the SP in AR.

## Abbreviations

Aldo-keto reductase (AKR); aldose reductase (AR); anion-binding pocket (ABP); AR inhibitors (ARIs); base of loop A subpocket (BLAS); caffeic acid phenethyl ester (CAPE); carboxylic acid (CA); carboxylic acid-containing inhibitor (CAI); gastrointestinal (GI); loop A subpocket (LAS); loop B subpocket (LBS); non-carboxylic acid-containing inhibitor (NCAI); non-steroidal anti-inflammatory drug (NSAID); Protein Data Bank (PDB); specificity pocket (SP).

## Appendix A

**Table A1:** Main features of AKR1B10 crystallographic structures.

PDB ID	Ligand Complexed to AKR1B10-NADP^+^	Molecular Formula of Ligand	Selectivity (Fold) ^#^	Pocket(s) Conformation	Trp112 in AKR1B10- or AR-Like Conformation	Loop A Subpocket (LAS) Occupation	Other Subpocket Occupation	Specificity Pocket (SP) in AR
1ZUA	tolrestat		AR (39)	Open SP Open LAS	AR-like	Trapped water molecule	-	Yes
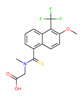
4GA8 *	sorbinil		AR (18)	Partially closed SP & LAS	AR-like	Trapped water molecule	-	No
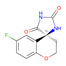
4GAB *	fidarestat		AR (1270)	Closed SP & LAS	AR-like	Trapped water molecule	-	No
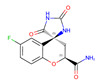
4GQ0	caffeic acid phenethyl ester		AKR1B10 (8)	Open SP Open LAS	AKR1B10-like	Yes	-	No
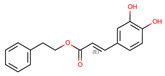
4GQG	apo	-	-	-	AKR1B10-like	Trapped water molecule	-	-
4I5X	flufenamic acid		AKR1B10 (54)	Partially closed SP & LAS	AKR1B10-like	No	-	No
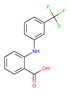
4ICC **	JF0064		AR (3.4)	Open loop B subpocket (LBS)	AR-like	No	LBS	No
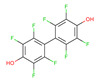
4JIH	epalrestat		AR (16)	Open SP Open LAS	AKR1B10-like	Yes? (partially modeled ligand in PDB)	-	No
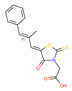
4JII	zopolrestat		AR (35)	Open SP Partially closed LAS	AR-like	Trapped water molecule	-	Yes
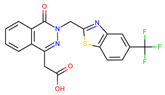
4WEV **	sulindac		AR (7.5)	Open SP Open LAS	AR-like (with interstitial water molecule)	Trapped water molecule	-	Yes
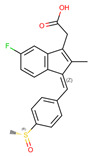
4XZL **	JF0049		AKR1B10 (8)	Open SP Open LAS	AR-like	Yes	-	No
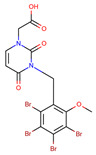
4XZM	apo (methylated)	-	-	-	AKR1B10-like	Trapped water molecule	-	-
4XZN **	apo AKME2MU (methylated)	-	-	-	AKR1B10-like	Trapped water molecule	-	-
5LIK **	MK181		AR (6)	Open SP Open LAS	AR-like (with interstitial water molecule)	Yes	-	Yes
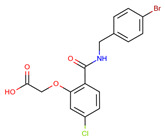
5LIU **	IDD388		AR (11)	Open SP Open LAS	AR-like (with interstitial water molecule)	Yes	-	Yes
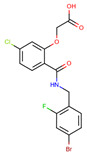
5LIW **	MK319		AR (23)	Open hyperextended SP Open LAS	AKR1B10-like	Trapped water molecule	-	Yes
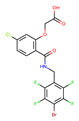
5LIX **	MK184		AKR1B10 (21)	Open extended SP Open LAS	AKR1B10-like	No	-	No
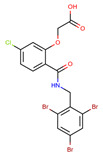
5LIY **	MK204		AKR1B10 (271)	Open hyperextended SP Open LAS	AKR1B10-like	Trapped water molecule	-	No
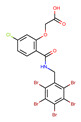
5M2F **	UVI2008		AKR1B10 (11)	Open SP Open LAS	AKR1B10-like	Yes	-	No
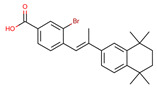
5Y7N	Androst-4-ene-3-beta-6-alpha-diol		AKR1B10 (136)	Open base of loop A subpocket (BLAS)	AKR1B10-like	No	BLAS	No
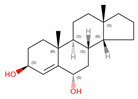

^#^ Selectivity fold of enzyme B versus enzyme A is defined as the ratio IC_50_ A/IC_50_ B, where A is the enzyme with high-er IC_50_ value (weaker inhibition) and B with lower IC_50_ value (more potent inhibition); * V301L mutant; ** methylated K125L/V301L mutant (AKME2MU); “-” not applicable.
